# Trauma, resilience and significant relationships: Sex differences in protective factors for military mental health

**DOI:** 10.1177/00048674241286818

**Published:** 2024-10-11

**Authors:** Lisa Dell, Kelsey Madden, Jenelle Baur, Alyssa Sbisa, Alexander McFarlane, Miranda VanHooff, Richard Bryant, Ellie Lawrence-Wood

**Affiliations:** 1 Phoenix Australia Centre for Posttraumatic Mental Health, Department of Psychiatry, The University of Melbourne, Carlton, VIC, Australia; 2 Discipline of Psychiatry, Adelaide Medical School, The University of Adelaide, Adelaide, SA, Australia; 3 Discipline of Psychiatry, Adelaide Medical School, The University of Adelaide and The University of South Australia, Adelaide, SA, Australia; 4 School of Psychology, University of New South Wales, Sydney, NSW, Australia

**Keywords:** Mental health, sex differences, military, veteran, subthreshold disorder

## Abstract

**Background:**

Military service is historically a male-dominated occupation, as such, the majority of research examining the development of mental disorder in Australian Defence Force members has had primarily male samples. While there have been mixed findings internationally regarding sex differences in rates of mental disorder and subthreshold symptoms among military personnel, across studies, the evidence tends to suggest that female military members are at least as likely as males to experience subthreshold mental health symptoms and have similar or higher rates of posttraumatic stress disorder despite the differences in roles during service. What is less understood is the impact of sex differences in symptom emergence over time and in predictors of clinical disorder.

**Method:**

The sample included a longitudinal cohort of Australian Defence Force members (*N* = 8497) surveyed at Time 1 (2010) and followed up at Time 2 (2015) on measures of anger, self-perceived resilience, trauma exposure, deployment exposure, suicidality, help-seeking, relationship satisfaction and mental health disorder symptoms. Outcomes included Subthreshold Disorder (above the optimal screening cut-off on the 10-item Kessler distress scale or posttraumatic stress disorder checklist) and Probable Disorder (above the epidemiological cut-off on the 10-item Kessler distress scale or posttraumatic stress disorder checklist).

**Results:**

Results found that while lifetime trauma exposure remained the strongest predictor of later probable disorder emergence among both males and females, for females specifically, self-reported resilience was also a significant protective factor. In contrast, being in a significant relationship at Time 1 was a protective factor against the development of subthreshold disorder in males.

**Conclusion:**

For the first time, sex differences in mental health symptom emergence over time have been explored in a large Australian cohort of military members. The capacity to adapt and bounce back after adversity emerged as a proactive factor against poor mental health for females in the military and could be addressed as part of routine skills training. Social support from significant relationship was particularly important for males’ mental health, suggesting that maintaining positive relationships and supporting military spouses and partners are critical for males’ mental health.

## Introduction

The number of females serving in militaries increases each year, and in the last decade, countries such as the United States and Australia have formally allowed females to serve in combat roles (Moore, 2017; Orme et al., 2016). There are currently differences in the types of roles that males and females hold within the military, which affords dissimilarities in exposure to traumatic stressors and may influence different mental health trajectories (Kelber et al., 2021). It is well established that posttraumatic mental health issues are prevalent among military populations (Bryant et al., 2019; Dell et al., 2023; Jones et al., 2020; Obuobi-Donkor et al., 2022) with clear evidence of the role of traumatic exposures conveying risk. However, research concerning the development of posttraumatic mental health issues in military populations has primarily focused on male samples, which is expected given the gender disparity in this group over the last several decades. The individual, social and economic burden of mental health issues (Bucci et al., 2019; Knapp and Wong, 2020; Nichter et al., 2020) combined with a greater number of females joining the military means that understanding sex differences regarding the impact of trauma and mental health trajectories is crucial.

Most prevalence studies including military members, veterans and the general population have reported higher rates of depression, anxiety and posttraumatic stress disorder (PTSD) in females than males (Parker and Brotchie, 2010; Shalev et al., 2019; Wisco et al., 2022); however, research into disorder chronicity between sexes has shown mixed findings to date (Dell et al., 2023; Jacobson et al., 2015; Kelber et al., 2021; van Zuiden et al., 2022). Sex differences in common mental health disorders have been theorised due to factors, such as gonadal hormones, gender roles, genetic predisposition and differences in trauma exposure (Christiansen and Berke, 2020; Kulkarni, 2023; Parker and Brotchie, 2010). Kelber et al. (2021) found that higher rates of new PTSD diagnoses in the US military females compared to males were not dependent on combat exposure, suggesting other forms of trauma (e.g. sexual) contributed to increased disorder rates in females. Sex differences have also been demonstrated in a UK military cohort, which found women reported increased odds of meeting the threshold for commonly reported mental disorder symptoms (Jones et al., 2020). Despite amounting research highlighting sex differences in common posttrauma mental health disorders, the question of how symptoms emerge over time and whether trajectories differ between sexes remains unclear.

Evidence to date suggests factors including combat exposure, increased number of deployments, and female gender enhance the likelihood of military personnel and veterans developing PTSD (Adler and Sipos, 2018; Xue et al., 2015), while the number of lifetime traumas has been found to be highly correlated with comorbid PTSD and depression, such that with each reported traumatic event, odds of comorbidity increases (Nichter et al., 2020). The Mental Health Prevalence and Wellbeing Transition Study report highlighted that female Australian Defence Force (ADF) members were more likely to experience anxiety disorders, panic attacks and PTSD than male ADF members (Van Hooff et al., 2018), which has also been observed in the general community (Farhane-Medina et al., 2022). In a longitudinal sample of veterans, occurrence of comorbid PTSD, depression and anxiety was higher than PTSD alone (Ginzburg et al., 2010), with evidence indicating that female gender increases risk for comorbidity among these disorders (Spinhoven et al., 2014).

In contrast to disorders, very little is known about the emergence of subthreshold symptoms posttrauma. An extensive longitudinal study of the mental health impacts during the initial years of military service in ADF personnel found that females were more likely than males to experience symptoms of psychological distress and posttraumatic stress during their first 3–4 years of service (Dell et al., 2023). This is particularly important to recognise given subthreshold symptoms can cause similarly high levels of distress and greater psychosocial dysfunction despite not meeting diagnostic criteria (Dickstein et al., 2013; Pietrzak et al., 2011). Increased understanding of symptom emergence could allow for earlier intervention, potentially derailing trajectories towards full disorder. In addition, given evident sex differences in the prevalence and presentation of disorders, such as PTSD and depression, and the growing representation of females in the military, exploration of sex in the emergence of posttraumatic mental health symptoms is increasingly necessary. Thus, this research aimed to examine sex differences in predictors of common mental disorder emergence over time in a large sample of Australian military personnel and veterans.

## Method

### Sample and procedure

The sample (*N* = 6501) included a longitudinal cohort of ADF members who participated in two studies: (Time 1) The Mental Health Prevalence and Wellbeing Study (MHPWS) component of the Military Health Outcomes Programme (MilHOP) (McFarlane et al., 2011), and (Time 2) the Transition and Wellbeing Research Programme (the Programme) (Van Hooff et al., 2018). The Programme followed up the MHPWS sample and comprised ADF members who transitioned from the ADF between 2010 and 2014 (‘Transitioned ADF’), and a sample of permanent full-time serving ADF personnel in 2015 (‘Regular ADF’). The current sample includes individuals who participated in both Time 1 and 2 and had complete data for the included variables. All participants were Regular ADF members at Time 1; however, by Time 2, some had transitioned from active service.

Data collection and linkage have been described in detail elsewhere (Bryant et al., 2019; McFarlane et al., 2011; Van Hooff et al., 2018); however briefly, at both time points, participants completed self-report surveys either online or in hard copy, which examined demographics, service, deployment history, mental health issues, psychological distress, physical health problems, wellbeing factors, pathways to care and occupational exposures. Measures of psychological and physical health remained the same between Time 1 and Time 2 where possible. Both the Programme (protocol no. E014/018) and MHPWS (protocol no. 574-09) had ethical approval from the Defence and Department of Veterans’ Affair Human Research Ethics Committees (DDVA HREC) and the Programme obtained mutual approval from Australian Institute of Health and Welfare Human Research Ethics Committee (AIHW HREC) (protocol no. EO2015/1/162).

**Figure 1.**
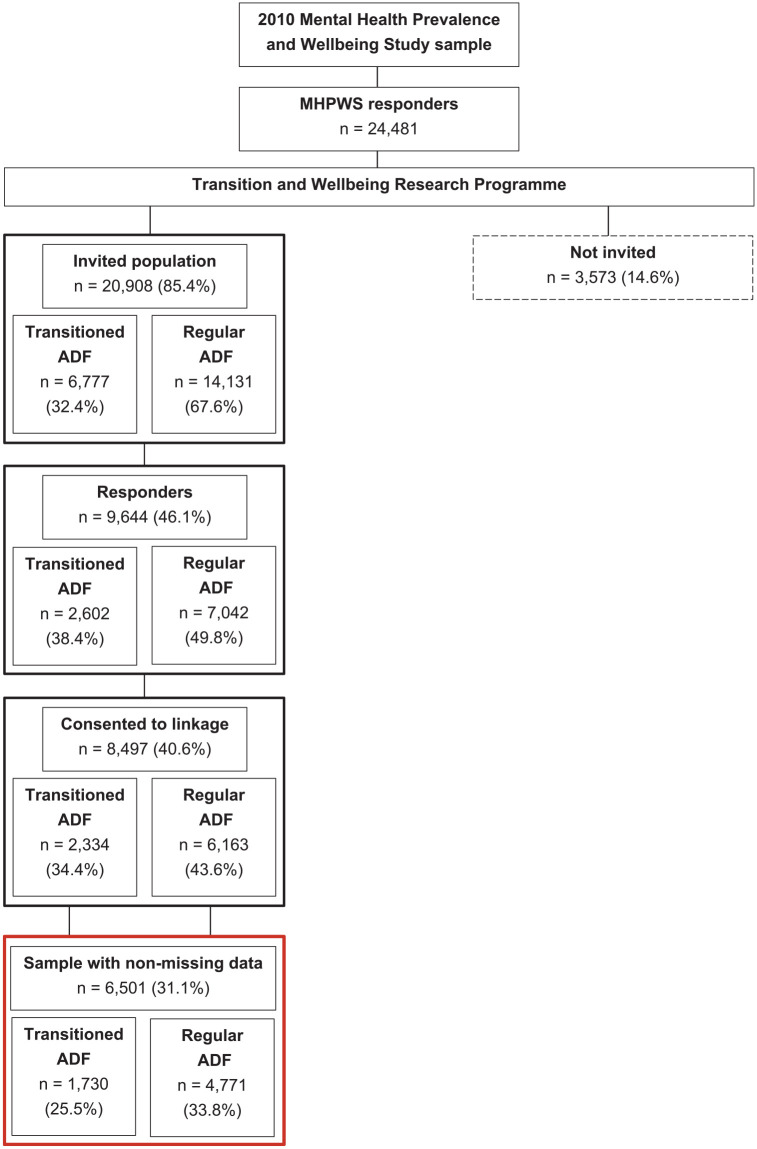
Survey response rates for the Transitioned ADF and the 2015 Regular ADF.

Figure 1 shows the survey response rates for the sample and the final complete dataset. All Time 1 responders, with the exception of those who did not consent to being contacted for future research, were invited to participate in Time 2. Responders to both time points were only included in the current sample if they consented to having their data points linked and had complete data for all variables of interest.

### Measures

#### Time 1 measures

##### Demographics

Sociodemographic characteristics included age, sex and relationship status. Service-related characteristics included transition status, ADF service branch (Army, Navy, Air Force), rank and length of service.

##### Relationship satisfaction

To assess satisfaction with marriage, participants were asked, ‘How satisfied are you with your marriage/relationship?’ Responses were recorded on a five-point scale from *extremely satisfied* to *extremely dissatisfied*, which was then dichotomised into ‘very dissatisfied/dissatisfied/neither’ and ‘satisfied/very satisfied’.

##### Help-seeking

Help-seeking was assessed using one item on the K10 + (‘In the past four [4] weeks, how many times have you seen a doctor or any other health professional about these feelings?’) (Kessler et al., 2002). Participants entered the number of times, which was then dichotomised into a yes/no variable indicating whether or not the participant had seen any health professionals in last month for psychological distress.

##### Suicidality

Participants were asked four questions about suicidal ideation, plans and attempts, and indicated whether they had experienced any of these items in the last 12 months. A dichotomous variable was created indicating whether the participant either reported any suicidality item or no suicidality items. Three of the items were adapted from the National Survey of Mental Health and Wellbeing (Australian Bureau of Statistics (ABS), 2007), and the final item was devised by researchers for use in the study (McFarlane et al., 2011).

##### Anger

Anger was assessed using the Dimensions of Anger Reactions 5-item scale (DAR-5) (Forbes et al., 2004). The DAR-5 examines anger frequency, intensity, duration, aggression and interference with social functioning over the last 4 weeks. Items are scored on a five-point Likert-type scale from 1 – *none of the time* to 5 – *all of the time*, generating a severity score ranging from 5 to 25, with higher scores indicating a higher frequency of anger. A cut-off score of ⩾ 12 was used to indicate problem anger. This scale has been used previously to assess Australian Vietnam veterans, and US Afghanistan and Iraq veterans, and shows strong unidimensionality, and high levels of internal consistency and criterion validity (Forbes et al., 2014a, 2014b).

##### Resilience

Resilience was assessed using two questions from the Connor–Davidson Resilience Scale (CD-RISC 2) (Vaishnavi et al., 2007). These items asked how often the participant felt they were able to adapt to change and tended to bounce back after hardship in the past 30 days. Statements are rated on a five-point scale from *not true at all* to *true nearly all the time*. Scores on these items were summed to create a total resilience score.

#### Time 2 measures

##### Lifetime trauma exposure

The measure to assess lifetime exposure to traumatic events was taken from the PTSD module of the Composite International Diagnostic Interview (CIDI) 3.0 (Haro et al., 2006). Participants were asked to indicate whether or not they had ever experienced 26 traumatic events, including ‘combat (military or organised non-military group)’, ‘being in a life-threatening automobile accident’ and ‘having someone close to you die unexpectedly’. The number of items endorsed was summed to create a total number of trauma types experienced by the participant and was then categorised into 0–1, 2–3, and >4 types.

##### Deployment exposures

Deployment exposures were assessed using items taken from the Middle East Area of Operations (MEAO) Census Study (Dobson et al., 2012). Participants reported how many times they had experienced a list of 12 deployment exposures during their military career. Response categories were 0 – ‘never’, 1 – ‘once’, 2 – ‘2–4 times’, 3 – ‘5–9 times’ and 4 – ‘more than 10 times’. Examples of events included ‘discharge of weapon in direct combat’ and ‘handled or saw dead bodies’. Traumatic deployment exposures were summed and then categorised into low (0–4), moderate (5–12) and high (13–48).

### Outcome variable

The outcome measure used was ‘disorder’, which used threshold classifications for both posttraumatic stress symptoms (measured using the PTSD Checklist civilian version [PCL-C]) and psychological distress (measured using the 10-item Kessler distress scale [K10]) consistent with the Bryant et al.’s (2019) longitudinal report of mental health changes in Australian military personnel.

PTSD symptoms were assessed using the PCL-C. The PCL-C comprises a 17-item self-administered questionnaire, which has been widely used for assessing PTSD symptoms over the past month. The PCL-C has excellent test–retest reliability and internal consistency, and has been used extensively in the context of population-based research (Weathers et al., 1993). A total symptom severity score was obtained by summing scores across items to give a score between 17 and 85, whereby higher scores indicate greater severity of PTSD symptoms. In accordance with ADF-specific cut-offs developed as part of the 2010 ADF MHPWS, a screening cut-off of 29 and an epidemiological cut-off of 53 were used (McFarlane et al., 2011).

Psychological distress was assessed using the K10. The K10 is a widely used and validated measure of non-specific psychological distress measured over the past 4 weeks (Kessler et al., 2003), which has demonstrated high levels of diagnostic accuracy, including Australian military populations (Searle et al., 2015). Respondents were instructed to rate the amount of time they had experienced in 1 of 10 emotional states during the last 4 weeks (e.g. tired for no good reason, nervous, hopeless, depressed). The 10 questions were scored from 1 to 5, whereby the respondent must indicate how often they have been feeling that way using one of the following response options: *all of the time* (5), *most of the time, some of the time, a little of the time* or *none of the time* (1). Scores for the 10 questions are then summed to give a total score from 10 to 50. In accordance with ADF-specific cut-offs developed as part of the 2010 ADF MHPWS, a screening cut-off of 17 and an epidemiological cut-off of 25 were applied (McFarlane et al., 2011).

The term ‘disorder’ in this paper is defined in relation to the scores on the K10 and PCL as follows:

- *No Disorder*: Below screening cut-off on both K10 and PCL;
- *Subthreshold Disorder*: Above the optimal screening cut-off on either the K10 or PCL, but below the optimal epidemiological cut-off on both K10 and PCL;
- *Probable Disorder*: Above the epidemiological cut-off on either the K10 or PCL.

### Statistical analysis

Analyses were conducted using SPSS v27. Frequencies and proportions of sample demographic and service characteristics were calculated separately for males and females. Multinomial logistic regression models were used to examine the univariate relationships between multiple potential predictors and disorder at Time 2 (no disorder, subthreshold, probable on either the K10 or PCL) among those who had no disorder at Time 1. This was conducted separately for males and females. Predictors included the following Time 1 variables: age, rank, service, length of service, relationship status, satisfaction with relationship, any suicide, problem anger, resilience, help-seeking in the last month and number of lifetime trauma types; and the following Time 2 variables: number of traumatic deployment exposure types and number of lifetime trauma types. Analyses examined the odds of having subthreshold disorder compared to no disorder at Time 2 or having probable disorder compared to no disorder at Time 2. Multivariate multinomial logistic regression models were then conducted separately for males and females using a fully saturated model, which included all variables with a *p*-value less than 0.20. The results are reported as odds ratios with 95% confidence intervals. A *p*-value less than 0.05 was considered significant.

## Results

Sociodemographic characteristics of the sample are presented in Table 1. The sample (*N* = 6501) consisted of 5514 males (mean age 39.2) and 987 females (mean age 34.5), primarily current serving ADF members rather than transitioned, and individuals predominantly served within the Army. Females served for an average of 11.2 years (SD 7.6) and males 16.2 years (SD 9.6).

**Table 1.** Demographic and service characteristics by sex.

	Females (*n* = 987)		Males (*n* = 5514)	
	*n*	%	*n*	%
**Transition status**				
Current	719	72.8	4052	73.5
Transitioned	268	27.2	1462	26.5
**Age (M, SD), years**	34.5	8.3	39.2	9.3
18–27	252	25.5	821	14.9
28–37	409	41.4	1615	29.3
38–47	250	25.3	2009	36.4
48–57	75	7.6	1019	18.5
>58	1	0.1	50	.9
**Service**				
Navy	259	26.2	1200	21.8
Army	373	37.8	2552	46.3
Air Force	355	36.0	1762	32.0
**Rank**				
Officer	443	44.9	2072	37.6
NCO	377	38.2	2829	51.3
Other ranks	167	16.9	613	11.1
**Length of service (M, SD)**	11.2	7.6	16.2	9.6
1 month–3.9 years	157	15.9	445	8.1
4–7.9 years	240	24.3	856	15.5
8–11.9 years	177	17.9	834	15.1
12–19.9 years	237	24.0	1201	21.8
>20 years	176	17.8	2178	39.5
**In a significant relationship**				
No	253	25.6	680	12.3
Yes	734	74.4	4834	87.7
**Satisfaction with relationship**				
N/A	216	21.9	496	9.0
Satisfied/very satisfied	690	69.9	4393	79.7
Very dissatisfied/dissatisfied/neither	81	8.2	625	11.3
**Seen professional for mental health in past 4 weeks**				
No	841	85.2	5047	91.5
Yes	146	14.8	467	8.5

Multinomial logistic regressions predicting subthreshold or probable disorder at Time 2 among males and females without disorder at Time 1 are presented in Tables 2 and 3, respectively. As expected, lifetime trauma exposure remains the strongest predictor of disorder emergence in both males and females. In females, compared to the reference category of ⩽ 1 trauma, ⩾ 4 lifetime traumas measured at Time 1 significantly predicted subthreshold and probable disorder; however, in males, lifetime traumas measured at Time 1 significantly predicted only subthreshold disorder.

**Table 2.** Factors predicting subthreshold or probable disorder at Time 2 in females with no disorder at Time 1.

Factor	Univariate		Multivariate	
	Subthreshold vs no disorder	Probable vs no disorder	Subthreshold vs no disorder	Probable vs no disorder
	OR (95% CI)	OR (95% CI)	OR (95% CI)	OR (95% CI)
**Demographics**				
Age	0.99 [0.96, 1.02]	1.02 [0.99, 1.06]	-	-
**Service factors**				
*Rank (ref: Officer)*				
NCO	1.49 [0.90, 2.45]	1.76 [0.94, 3.28]	0.88 [0.45, 1.72]	1.69 [0.79, 3.62]
Other ranks	1.98 [1.06, 3.70]*	1.69 [0.73, 3.90]	2.06 [0.99, 4.28] †	1.96 [0.74, 5.16]
*Service branch (ref: Air force)*				
Navy	0.90 [0.50, 1.61]	1.00 [0.48, 2.12]	-	-
Army	0.93 [0.55, 1.55]	1.10 [0.58, 2.11]	-	-
*Length of service (M, SD) (ref: >20)*				
1 month–3.9 years	0.89 [0.40, 1.94]	0.55 [0.19, 1.57]	-	-
4–7.9 years	1.04 [0.52, 2.10]	0.66 [0.27, 1.63]	-	-
8–11.9 years	1.17 [0.56, 2.45]	1.17 [0.49, 2.77]	-	-
12–19.9 years	0.96 [0.47, 1.96]	0.93 [0.40, 2.16]	-	-
**Relationship status and satisfaction**				
In a significant relationship (ref: No)				
Yes	0.89 [0.53, 1.50]	0.84 [0.44, 1.60]	-	-
*Satisfaction (ref: Very dissatisfied/dissatisfied/neither)*				
Satisfied/very satisfied	0.67 [0.24, 1.88]	0.37 [0.13, 1.05]	-	-
**Suicidality, anger and resilience (ref: No)**				
*Any suicide*				
Yes	0.66 [0.15, 2.97]	3.08 [1.07, 8.93]*	1.28 [0.26, 6.31]	2.12 [0.51, 8.85]
*Problem anger*				
Yes	2.05 [0.52, 8.07]	1.16 [0.14, 9.6]	-	-
*Resilience*	0.75 [0.61, 0.91]**	0.73 [0.57, 0.93]**	0.73 [0.58, 0.93]*	0.66 [0.51, 0.87]**
**Help-seeking**				
*Seen any health professional in last month for feelings of psychological distress (ref: No)*				
Yes	1.62 [0.70, 3.73]	0.66 [0.15, 2.88]	-	-
**Lifetime trauma**				
*Number of lifetime trauma types (ref: 0–1)*				
2–3	1.23 [0.62, 2.44]	1.29 [0.56, 2.98]	1.34 [0.66, 2.71]	1.33 [0.56, 3.15]
>4	2.57 [1.33, 4.97]**	3.07 [1.41, 6.68]**	2.52 [1.25, 5.07]*	2.8 [1.23, 6.36]*
**Deployment exposures**				
*Number of deployment exposures (ref: Low)*				
Moderate	1.52 [0.89, 2.59]	1.57 [0.82, 3.03]	9.11 [0.41, 2.04]	1.69 [0.75, 3.80]
High	2.94 [1.51, 5.74]**	2.13 [0.87, 5.22]	2.64 [1.00, 7.00]	1.35 [0.35, 5.29]

Only statistically significant and trending predictors within the univariate analysis were included in the multivariate model.

† *p* < 0.06, *p < 0.05, **p < 0.01, ***p *<* 0.001.

**Table 3.** Factors predicting subthreshold or probable disorder at Time 2 in males with no disorder at Time 1.

Factor	Univariate		Multivariate	
	Subthreshold vs no disorder	Probable vs no disorder	Subthreshold vs no disorder	Probable vs no disorder
	OR (95% CI)	OR (95% CI)	OR (95% CI)	OR (95% CI)
**Demographics**				
Age	1.00 [0.99, 1.01]	0.99 [0.98, 1.00] ^†^	1.00 [0.98, 1.02]	1.01 [0.99, 1.03]
**Service factors**				
*Rank (ref: Officer)*				
NCO	1.51 [1.24, 1.85]***	1.77 [1.37, 2.29]***	2.00 [1.48, 2.70]***	1.85 [1.30, 2.62]**
Other ranks	1.47 [1.07, 2.03]*	2.26 [1.56, 3.26]***	1.95 [1.21, 3.14]**	1.93 [1.13, 3.32]*
*Service (ref: Air force)*				
Navy	1.55 [1.19, 2.03]**	1.29 [0.92, 1.80]	1.58 [1.10, 2.27]*	1.33 [0.84, 2.11]
Army	1.83 [1.47, 2.28]***	1.79 [1.37, 2.34]***	0.98 [0.69, 1.38]	1.43 [0.95, 2.14]
*Length of service (M, SD) (ref: >20)*				
1 month–3.9 years	1.03 [0.72, 1.47]	1.47 [0.99, 2.19]	0.92 [0.54, 1.58]	1.19 [0.67, 2.13]
4–7.9 years	1.08 [0.82, 1.43]	1.58 [1.15, 2.18]**	0.89 [0.54, 1.45]	1.21 [0.71, 2.09]
8–11.9 years	1.27 [0.96, 1.66]	1.16 [0.81, 1.66]	1.16 [0.72, 1.87]	0.75 [0.40, 1.39]
12–19.9 years	1.21 [0.95, 1.54]	1.19 [0.88, 1.62]	1.10 [0.74, 1.63]	1.17 [0.74, 1.86]
**Relationship status and satisfaction**				
*In a significant relationship (ref: No)*				
Yes	0.78 [0.59, 1.03]	0.73 [0.52, 1.01]	0.69 [0.50, 0.96]*	0.82 [0.55, 1.21]
*Satisfaction (ref: Very dissatisfied/dissatisfied/neither)*				
Satisfied/very satisfied	0.82 [0.59, 1.13]	0.91 [0.6, 1.38]	-	-
**Suicidality, anger and resilience (ref: No)**				
*Any suicide*				
Yes	1.49 [0.80, 2.79]	1.85 [0.92, 3.71]	0.40 [0.09, 1.77]	2.02 [0.79, 5.20]
*Problem anger*				
Yes	3.04 [1.8, 5.14]***	2.75 [1.45, 5.2]**	2.46 [1.18, 5.13]*	1.42 [0.52, 3.91]
*Resilience*	0.88 [0.81, 0.94]***	0.95 [0.86, 1.04]	0.97 [0.87, 1.09]	1.08 [0.94, 1.25]
**Help-seeking**				
*Seen any health professional in last month for feelings of psychological distress (ref: No)*				
Yes	2.22 [1.43, 3.43]***	1.39 [0.75, 2.59]	1.48 [0.68, 3.21]	1.33 [0.49, 3.58]
**Lifetime trauma**				
*Number of lifetime trauma types (ref: 0–1)*				
2–3	1.11 [0.76, 1.60]	1.22 [0.81, 1.84]	1.06 [0.73, 1.55]	1.14 [0.75, 1.74]
>4	2.43 [1.80, 3.27]***	1.85 [1.30, 2.63]**	1.87 [1.35, 2.58]***	1.35 [0.92, 1.98]
**Deployment exposures**				
Number of deployment exposures (ref: Low)				
Moderate	1.60 [1.26, 2.05]***	1.34 [0.99, 1.81]	1.35 [0.95, 1.92]	0.99 [0.64, 1.52]
High	3.11 [2.50, 3.86]***	2.60 [2.00, 3.37]*	2.90 [2.06, 4.06]**	2.53 [1.71, 3.73]**

Only statistically significant and trending predictors within the univariate analysis were included in the multivariate model.

† *p* < 0.06, *p < 0.05, **p < 0.01, ***p < 0.001.

Additional predictors for subthreshold disorder in males included service branch, self-reported problem anger and number of traumatic deployment exposures. However, the traumatic deployment exposure variable was approaching significance for females (*p* = 0.051). Compared to the reference category of Air Force, males in the Navy had 1.58 greater odds of developing subthreshold disorder, and males with self-reported problem anger at Time 1 had 2.46 times greater odds of developing subthreshold disorder at Time 2. In male participants, a high number of deployment exposures (⩾ 13) recorded at Time 2 significantly predicted both subthreshold and probable disorder.

In males, reporting a significant relationship at Time 1 was a protective factor against the development of subthreshold disorder, while in females, self-reported resilience was a significant protective factor against the development of both subthreshold and probable disorder. Likelihood ratio tests revealed that final models were statistically significant (*p* < 0.001) for both females and males, indicating that the full model was able to statistically significantly predict disorder; however, both models were weak. For females, the multinominal logistic regression model explained 12% (Nagelkerke’s *R*^2^) of the variance in symptoms. For males, the multinominal logistic regression model explained 11% (Nagelkerke’s *R*^2^) of the variance in symptoms.

## Discussion

This paper has provided a unique insight into the impact of sex differences on the emergence of mental health disorder in Australian military members over time. With the vast majority of Australian military mental health research focused on male populations, it is critical to ensure that we use large study cohorts to examine sex differences in this occupational group.

Lifetime trauma, resilience, anger and relationship status emerged as key factors in this study and were found to affect males and females differently. As expected, lifetime trauma was the strongest predictor of disorder among both males and females, consistent with previous posttraumatic research (Brownlow et al., 2018; Nichter et al., 2020; Reger et al., 2019). However, importantly and more specifically, the current study found that four or more traumas predicted both subthreshold and probable disorder for females, whereas for males, two or more lifetime traumas predicted only subthreshold disorder. This suggests the cumulative effect of trauma on mental health may impact males and females differently and that it is important to track lifetime trauma and understand the unique differences for females and males in terms of accumulation. It is important to note, however, that females are more likely to experience interpersonal trauma (such as sexual assault) (Olff, 2017; Street et al., 2013), which may contribute to the cumulative effect of trauma. Our finding is consistent with large longitudinal research in Australian military personnel, where experiencing four or more traumas increased the risk of disorder emergence (Dell et al., 2019) but expands on previous literature by unpacking the impact of sex differences.

Self-reported resilience emerged as the main protective factor against subthreshold and probable disorder among females. This finding expands on previous research that did not consider sex-specific differences (Cusack et al., 2016; Dhungana et al., 2022; Lee et al., 2017; van der Meulen et al., 2020). The finding that the capacity to adapt after adversity may be particularly important for the mental health of females and has important clinical implications. It may be that female military personnel will uniquely benefit from targeted prevention and early intervention training that aims to build the skills necessary to adapt in challenging situations. This kind of training may increase the likelihood of rapid adaptive responses to adversity and prevent symptom progression for females (Ludolph et al., 2019; Nehra et al., 2019; Watson, 2019). Social support has been clearly established as a protective factor against poor mental health, including support from a spouse/partner (Dell et al., 2023; Downward et al., 2022; Harandi et al., 2017). For the males in this study, being in a significant relationship was protective against later development of subthreshold disorder – a finding which did not emerge within the female cohort. One explanation for significant relationships playing an important role in protecting the mental health of males relates to the varied perception of social support between sexes (Stronge et al., 2019). Males are more likely to perceive a higher degree of social support when they are in a significant relationship compared to females (Stronge et al., 2019). Comparably, females are more likely to have broad social support networks which include friendships (Antonucci et al., 2014; Stronge et al., 2019), thus defining their social support network more broadly than men. The tendency for men to rely on spouses for social support may relate to masculinity norms in heterosexual relationships, whereby men are less likely share emotions with other men and instead rely on women for emotional support (McKenzie et al., 2018). As such, it is critical that males in the military have a healthy relationship with their spouse for whom they rely on for support, and that female spouses are also supported to ensure this reliance does not place high burden on them (Stronge et al., 2019). Importantly, while the current research indicated relationships were protective against progression towards subthreshold disorder for men, findings did not extend to probable disorder, suggesting there may be a threshold for number of traumas or chronicity of symptoms which, once surpassed, impacts the extent to which relationships protect against progression towards probable disorder. This finding warrants further investigation in future research.

A second factor unique to males that emerged was the experience of problematic anger, with anger found to be a predictor of later development of both subthreshold and probable disorder. This finding indicates that problematic anger may be an early indicator of mental health change among military men. Several explanations have been posed to explain sex differences in anger, including men’s psychosocial factors (Dmitrieva et al., 2011). This finding supports the notion that anger is an early indicator of mental health challenges among male military populations, and targeting and reducing anger responses may improve mental health (Cash et al., 2018), while potentially preventing further mental health decline. Sex differences in anger are well reported in the literature (Fahlgren et al., 2022; Kannis-Dymand et al., 2019; Repple et al., 2018) with more recent research beginning to investigate biological underpinnings of sex differences in anger responses (Long et al., 2022). To our knowledge, the current research is the first to report sex differences in anger as a predictor of later mental health decline. Other predictors of subthreshold disorder in males included service branch and number of traumatic deployment exposures. Traumatic deployment also predicted probable disorder, which has been well established within the literature (Highfill-McRoy et al., 2022; Pietrzak et al., 2013; Xue et al., 2015).

Many of the key factors identified as predicting mental health disorder emergence are modifiable factors, which have the potential to be targeted with training programmes or early interventions. Several resilience interventions currently exist (Chmitorz et al., 2018); however, further research is needed to ensure interventions are suitable for females. Similarly, anger may be targeted through interventions; however, identifying anger in oneself and seeking help remain a challenge among military populations (Forbes et al., 2013). In fact, the current data found males were less likely than females to seek help, and seeking help was a significant predictor of later development of subthreshold mental health symptoms among males and not females. Continuing to build awareness around the importance of seeking help is vitally important for preventing mental health challenges in military personnel. Importantly, the data presented here help to uncover potential differences in what buffers against mental health symptom development, which ultimately highlights the importance of considering sex differences in mental health research.

The current study is not without methodological limitations including the use of self-report data. Furthermore, the outcome variable used was a combination of scores on measures of depression and PTSD, which does not enable findings to be interpreted as specific to either disorder. Finally, a large proportion of the dataset was male which may have contributed to fewer significant factors being identified for females.

## Conclusion

Regardless of limitations, the current findings provide some evidence for there being sex differences in predictors of later probable disorder emergence. Future research should continue to explore sex differences in predictors of mental health disorder emergence to continue building knowledge of sex-specific factors that help protect against later development of symptoms for males and females.
